# KDM4B plays an important role in mitochondrial apoptosis by upregulating HAX1 expression in colorectal cancer

**DOI:** 10.18632/oncotarget.11077

**Published:** 2016-08-05

**Authors:** Haijie Li, Xi Yang, Guihua Wang, Xiaolan Li, Deding Tao, Junbo Hu, Xuelai Luo

**Affiliations:** ^1^ Cancer Research Institute, Tongji Hospital, Huazhong University of Science and Technology, Wuhan, China

**Keywords:** KDM4B, HAX1, mitochondrial apoptosis, colorectal cancer

## Abstract

Histone methyltransferases and demethylases regulate transcription by altering the epigenetic marks on histones in tumorigenesis. Members of the histone lysine(K)-specific demethylase 4 (KDM4) family are dysregulated in several types of cancer. Here, we report a novel role for KDM4B in mitochondrial apoptosis. In this study, we demonstrate that KDM4B is overexpressed in colorectal cancer (CRC) tissues. Decreased expression of KDM4B significantly promoted apoptosis of CRC cell lines. Moreover, our data indicate that HAX1 is required for KDM4B-mediated mitochondrial apoptosis. The transcription of *HAX1* was directly activated by KDM4B. We also show that HAX1 is overexpressed in CRC tissues and is positively correlated with KMD4B expression. Collectively, we demonstrate that KDM4B may play an important role in mitochondrial apoptosis and represent a potential therapeutic cancer target in CRC.

## INTRODUCTION

The role of epigenetic abnormalities in the initiation and progression of cancers is well recognized [1]. Histone methylation, one such epigenetic modification, plays an important role in the functional regulation of gene expression, by activating or repressing transcription [2]. This modification takes place on lysine residues, and prior to the discovery of histone demethylases, was thought to be essentially irreversible [3]. Histone demethylases remove methyl groups from the lysine residues of histone tails, thereby regulating the transcriptional activity of target genes [4, 5].

KDM4B, also known as JMJD2B, is a recently identified member of the histone demethylase KMD4 family [6]. Previous studies have shown that KDM4B is capable of removing the trimethyl group from histone H3 lysine 9, on pericentric heterochromatin and euchromatin [7]. Recent studies have also indicated a role for KDM4B in transcription. For example, KDM4B can influence H3K9 methylation in hormonally responsive breast cancer [8], and function as an estrogen receptor (ER) co- factor [9, 10]. Additionally, KDM4B has been shown to harbor HIF binding sites in their promoter sequencesin hypoxic conditions [11]. However, the role of KDM4B in apoptosis remains unclear.

Apoptosis represents one of the most important forms of cell death in multicellular organisms, and is typically dysregulated in human cancers [12]. In mammals, apoptosis may occur via two main pathways: the death receptor pathway (extrinsic apoptotic pathway) and the mitochondrial pathway (intrinsic apoptotic pathway) [13]. It is well established that mitochondria are key decoding stations of the apoptotic process, and play a fundamental role in triggering apoptotic cell death [14]. As the central organelle of the intrinsic apoptotic pathway, mitochondria are reservoirs of proapoptotic factors. Upon apoptotic stimulation, the mitochondrial permeability transition pore is opened and mitochondrial membrane potential (MMP) is dissipated, culminating in the release of proapoptotic proteins [15, 16]. Thus, the collapse of MMP represents the early stage of mitochondrial apoptosis.

In this study, we demonstrate that KDM4B is overexpressed in colorectal cancer and is significant for the mediation of mitochondrial apoptosis in colorectal cells. We suggest that KDM4B may represent a potential therapeutic cancer target in CRC.

## RESULTS

### Overexpression of KDM4B in clinical colorectal cancers

**Figure 1:**
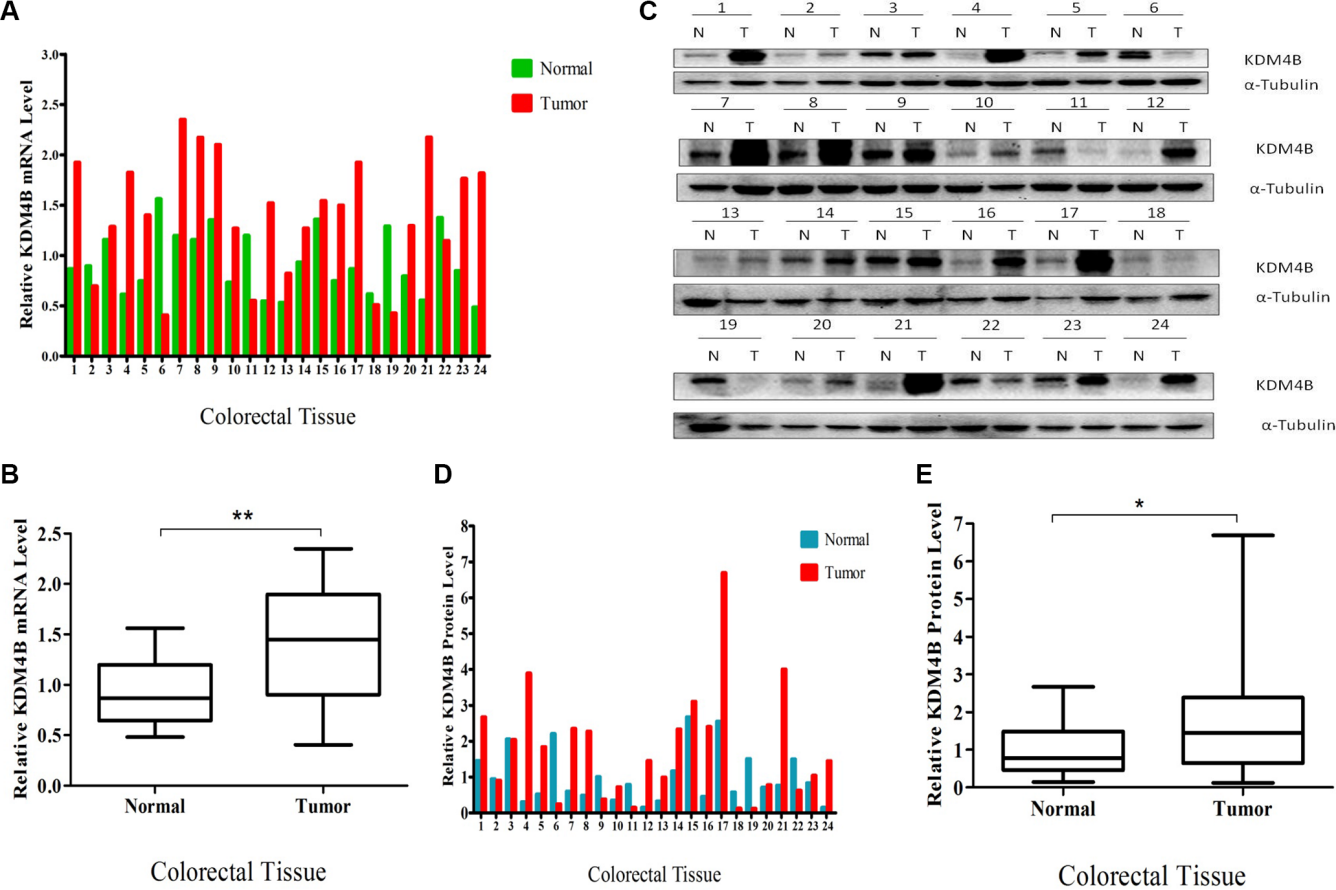
Overexpression of KDM4B in clinical colorectal cancer (**A**) Quantitative real-time PCR of *KDM4B* was performed using 24 pairs of colorectal tissues including 24 colorectal tumor tissues and 24 corresponding colorectal normal tissues. (**B**) Quantitative real-time PCR results are shown in the box-whisker plot. Student's *t* test (*P* < 0.01). (**C**) Western blot analysis of KDM4B protein expression in 24 pairs of colorectal tissues. (**D**) Quantification of KDM4B protein in colorectal tissues. Band intensities were measured using Image J and normalized to α-tubulin. (**E**) Quantification of KDM4B is shown by box-whisker plot. Student's *t* test (*P* < 0.05).

We first measured *KDM4B* mRNA levels in 24 primary colorectal tumor samples and corresponding adjacent normal colorectal tissues by quantitative RT- PCR. *KDM4B* mRNA levels were significantly higher in tumor samples than normal tissues (*P* = 0.0011, Figure 1A and 1B), and KDM4B protein was also overexpressed in CRC specimens, as assessed by western blot (Figure 1C). Quantification and statistical analysis of western blot data using Image J v1.48 software confirmed that KDM4B protein levels were significantly higher in tumors than in normal tissues (Figure 1D and 1E, *P* < 0.05). In order to further validate our results, we performed the NCBI Gene Expression Omnibus (GEO) dataset parameters to disclose the KDM4B expression in normal tissues and tumors. It was gratifying that the analysis result was consistent with our findings (Supplementary Figure S1A). Taken together, these results show that KDM4B levels are significantly higher in colorectal cancer tissues than in corresponding non-neoplastic tissues.

### KDM4B regulates cell apoptosis

**Figure 2:**
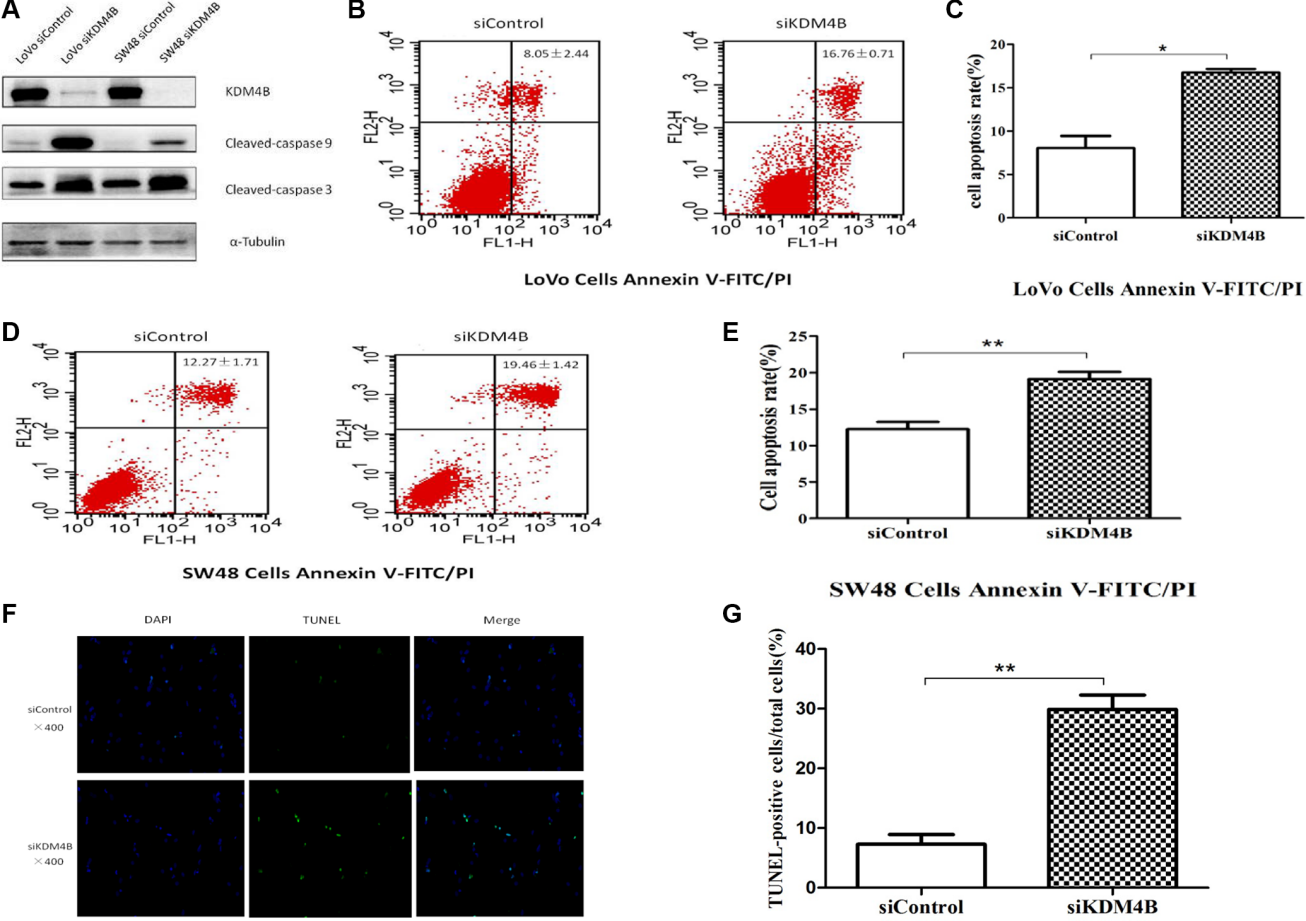
KDM4B expression is closely associated with cell apoptosis (**A**) Effect of KDM4B knockdown on levels of cleaved-caspase 9 and cleaved-caspase 3. LoVo and SW48 cells were treated with siControl or siKDM4B (1#) for 72 h, then cleaved-caspase 9 and cleaved-caspase 3 levels were examined by western blot. (**B** and **C**) Knockdown of KDM4B induces significant apoptosis in LoVo cells by Annexin V-FITC/PI staining analysis. LoVo cells were treated with siControl (8.05 ± 2.44) or siKDM4B (1#,16.76 ± 0.71) for 72 h, then cell apoptosis was tested by Annexin V-FITC/PI staining analysis.(C, *P* < 0.05) (**D** and **E**) Knockdown of KDM4B induces significant apoptosis in SW48 cells by Annexin V-FITC/PI staining analysis. SW48 cells were treated with siControl (12.27 ± 1.71) or siKDM4B (1#, 19.46 ± 1.42) for 72 h, then cell apoptosis was tested by Annexin V-FITC/PI staining analysis. (E, *P* < 0.01) (**F** and **G**) TUNEL assay. LoVo cells were treated with siControl (6.26 ± 1.38) and siKDM4B (1#, 28.91 ± 2.76) for 72 h and TUNEL assays were performed as described in the Methods section. (G, *P* < 0.001) Blue: nucleus, Green: the fractured DNA.

Previous studies have shown that KDM4B may promote growth in bladder cancer, lung cancer [17], and gastric cancer [18]. Deregulation of cell apoptosis was shown to be the major cause of tumor growth, so we next investigated the role of KDM4B in CRC cell apoptosis. To explore the function of KDM4B in CRC cell apoptosis, we depleted KDM4B expression in LoVo and SW48 cells. As shown in Supplementary Figure S1B, S1C and S1D, treatment of cells with siRNA specifically targeting KDM4B (Supplementary Table S3) led to significant inhibition of KDM4B expression. We next analyzed the activation pattern of caspase 9 and caspase 3 (cleaved-caspase 9 and cleaved-caspase 3) following treatment with KDM4B siRNA. KDM4B knockdown led to a significant increase in the level of cleaved-caspase 9 and cleaved-caspase 3 (Figure 2A). In addition, knockdown of KDM4B in LoVo and SW48 cells led to a significant increase in apoptosis, assessed by Annexin V-FITC/PI staining (Figure 2B–2E, Supplementary Figure S2A and S2B). These results indicate that knockdown of KDM4B promotes cell apoptosis. Moreover, KDM4B knockdown led to a significant increase in apoptotic cells, assessed by TUNEL assay (Figure 2F and 2G, *P* < 0.001).

To further illustrate the effect of KDM4B on cell apoptosis, the effect of overexpressing KDM4B on apoptosis was also investigated. Transfection of LoVo and SW48 cells with an KDM4B expression vector (Supplementary Figure S1E and S1F) did not lead to significant changes in cleaved-caspase 9/3 or Annexin V-FITC/PI staining (Supplementary Figure S2C, S2D and S2E), likely because of the high level of endogenous KDM4B expression in these cells.

Taken together, these results demonstrate that KDM4B silencing promotes cell apoptosis, robustly demonstrating a close association between KDM4B and cell apoptosis.

### KDM4B plays an essential role in mitochondrial apoptosis by targeting HAX1

**Figure 3:**
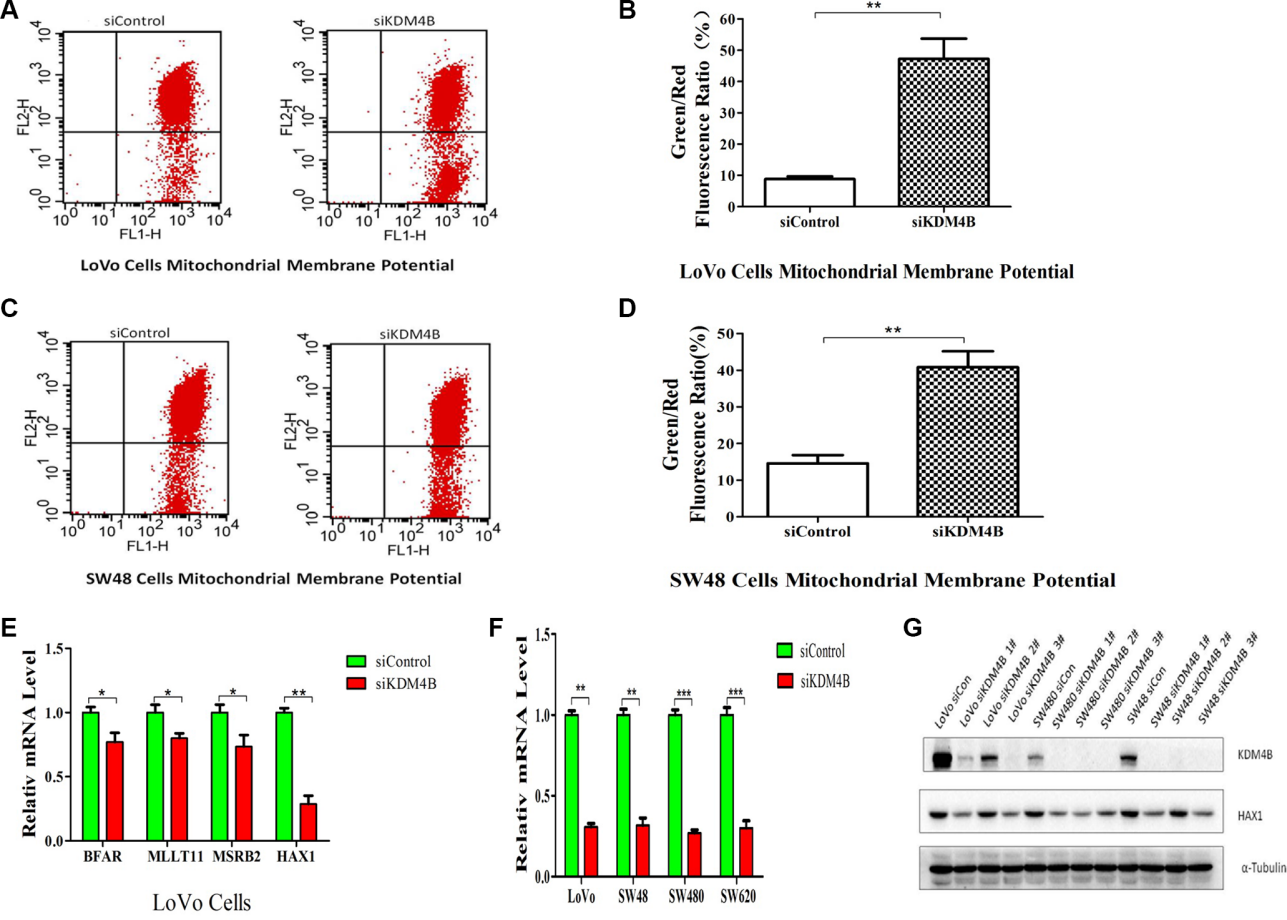
KDM4B plays an essential role in mitochondrial apoptosis (**A** and **B**) KDM4B knockdown led to a decrease in mitochondrial membrane potential in LoVo cells. The mitochondrial membrane was stained with JC-1, which exists as a green fluorescent monomer at low membrane potential and as red fluorescent aggregates at high membrane potential. The ratio of green to red fluorescence of JC-1 reflects the ratio of apoptosis. LoVo cells were treated with siControl (8.83 ± 1.47) or siKDM4B (1#, 47.28 ± 11.15) for 72 h, then mitochondrial membrane potential was tested by JC-1 staining analysis.(B, *P* < 0.01). (**C** and **D**) KDM4B knockdown led to a decrease in mitochondrial membrane potential in SW48 cells. SW48 cells were treated with siControl (14.60 ± 3.87) or siKDM4B (1#, 40.84 ± 7.52) for 72 h, then mitochondrial membrane potential was tested by JC-1 staining analysis.(D, *P* < 0.01) (**E**) Expression levels of *BFAR*, *MLLT11*, *MSRB2,* and *HAX1* in LoVo cells following treatment with siKDM4B for 72 h. mRNA levels were analyzed by quantitative RT- PCR.(*P < 0.05; **P < 0.01) (**F** and **G**) Expression levels of HAX1 in colorectal cancer cells (LoVo, SW48, SW480, SW620) following treatment with siKDM4B for 72 h by quantitative RT-PCR (siKDM4B 1#) and western blot (siKDM4B 1, 2, 3#). (**P < 0.01; ***P < 0.001) All qRT-PCR data were normalized to 18s ribosomal RNA, and α-tubulin was used as a loading control for western blots.

Mitochondria are the key decoding stations of the apoptotic process and play a fundamental role in triggering apoptotic cell death [14]. To clarify the mechanisms underlying KMD4B-regulated apoptosis, we investigated whether KDM4B was involved in mitochondrial apoptosis pathways. The collapse of mitochondrial membrane potential (MMP) coincides with the opening of mitochondrial permeability transition pores, leading to the release of cytochrome c into the cytosol, which in turn triggers other downstream events in the apoptotic cascade [19]. We therefore analyzed MMP using JC-1, in CRC cells treated with KDM4B siRNA. KDM4B knockdown led to a significant decrease in MMP in LoVo cells and SW48 cells (Figure 3A–3D), indicating that KDM4B may be involved in mitochondrial apoptosis.

Recent microarray studies investigating the role of KDM4B in carcinogenesis, indicate that HAX1 may be a target of KDM4B [17]. Analysis of these microarray results identified several apoptosis-related genes, including *VPS53*, *HAX1*, *PPP6C*, *BFAR*, *MLLT11*, and *MSRB2*. We next confirmed regulation of these genes by KDM4B individually by quantitative RT-PCR (Figure 3E and Supplementary Figure S3A). Of these genes, *HAX1* expression was notably downregulated following KMD4B knockdown and upregulated following KDM4B overexpression. Previously, we showed that HAX1 was critical for conserving MMP and for protecting cells from mitochondrial apoptosis [20]. We therefore hypothesized that HAX1 may play a role in KDM4B-induced apoptosis. To confirm this hypothesis, we further analyzed HAX1 expression in cells following KDM4B knockdown or overexpression in additional colorectal cancer cell lines. HAX1 expression was notably downregulated following KMD4B knockdown (Figure 3F and 3G), and were also increased following KDM4B overexpression in colorectal cancer cell lines (Supplementary Figure S3B and S3C). We also analyzed the expression of Bcl-2 and Bax, critical regulators of mitochondrial apoptosis [16]. In contrast to HAX1, however, KDM4B knockdown had no clear effect on Bcl-2 levels and had a minimal effect on Bax (Supplementary Figure S3D). These results indicate that *HAX1*, an anti-apoptosis gene, is a prominent target gene of KDM4B.

### KDM4B transcriptionally activates HAX1 expression and inhibits methylation of H3K9me3 at the *HAX1* promoter region

**Figure 4:**
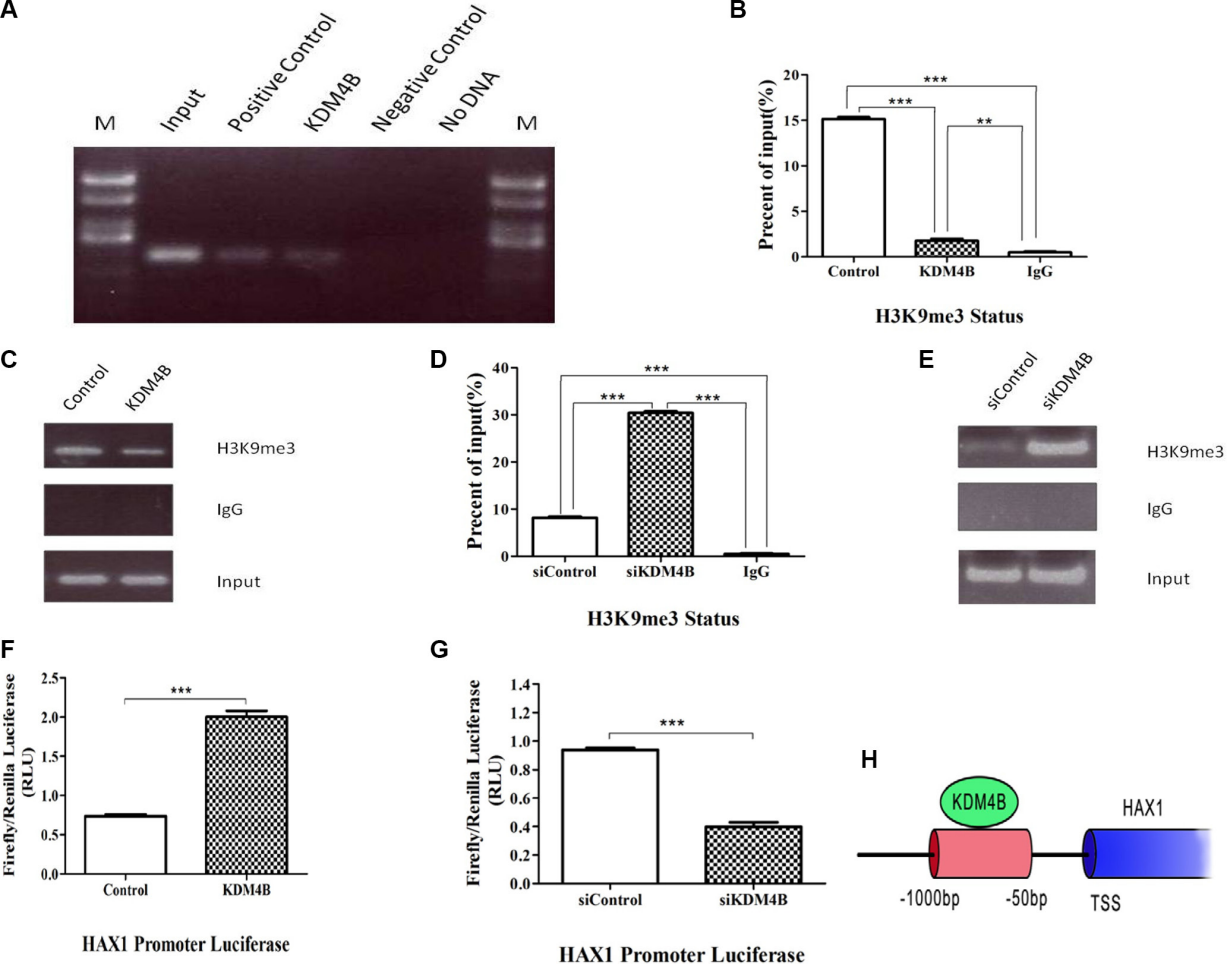
KDM4B transcriptionally activates HAX1 expression via demethylation of H3K9me3 at the promoter region (**A**) Chromatin immunoprecipitation (ChIP) assay for KDM4B at the *HAX1* promoter. ChIP-PCR of samples were analyzed by agarose gel electrophoresis in LoVo cells. Positive control: RNA polymerase II, Negative control: normal mouse IgG. (**B**, **C**, **D**, and **E**) ChIP assay for H3K9me3 at the *HAX1* promoter. LoVo cells were transfected with KDM4B plasmid for 48 h (C and D) or treated with siKDM4B for 72 h (E and F) prior to ChIP analysis using an anti-H3K9me3 antibody, and subsequent analysis at the *HAX1* promoter. (**F** and **G**) LoVo cells were transfected with KDM4B plasmid (G) or treated with siKDM4B (1#,(**H**)) and the *HAX1* promoter plasmid. Relative luciferase activity was assayed. H. A schematic of the *HAX1* promoter region. The red region indicates PCR-amplified regions. TSS, transcription start site.

KDM4B has been shown to demethylate histone H3 at lysine 9 (H3K9) at gene promoters, and is capable of removing tri-methyl or di-methyl marks. [7, 21]. Based on our finding that the anti-apoptosis gene, *HAX1*, is an important KDM4B target, we therefore focused on understanding the role of KDM4B in mediating *HAX1* expression. ChIP assays were performed to investigate whether the *HAX1* promoter was occupied by KDM4B. KDM4B was localized at the promoter of *HAX1* in LoVo cells (Figure 4A and Supplementary Figure S4A), and KDM4B knockdown led to an increase in H3K9me3 levels at the *HAX1* promoter. Correspondingly, KDM4B overexpression led to a decrease in H3K9me3 levels at the *HAX1* promoter (Figure 4B–4E). We next examined the ability of KDM4B to regulate the activity of the *HAX1* promoter using luciferase reporter assays. KDM4B knockdown significantly repressed *HAX1* promoter activity, while KDM4B overexpression significantly enhanced promoter activity (Figure 4F and 4G). Furthermore, we deleted the JmjC domain (Location:176-292, pfam02373) of KDM4B and constructed a KDM4B “demethylase-dead” plasmid (ΔKDM4B, Supplementary Figure S4B). Then we repeated the luciferase reporter assays and the enhancement was disappeared (Supplementary Figure S4C). The ChIP assays showed the similar results (Supplementary Figure S4D and S4E). Taken together, these results indicate that KDM4B directly activates HAX1 expression and regulates methylation of histone H3 at lysine 9 at the *HAX1* promoter (Figure 4H).

### KDM4B regulates mitochondrial apoptosis in a partly HAX1-dependent manner

**Figure 5:**
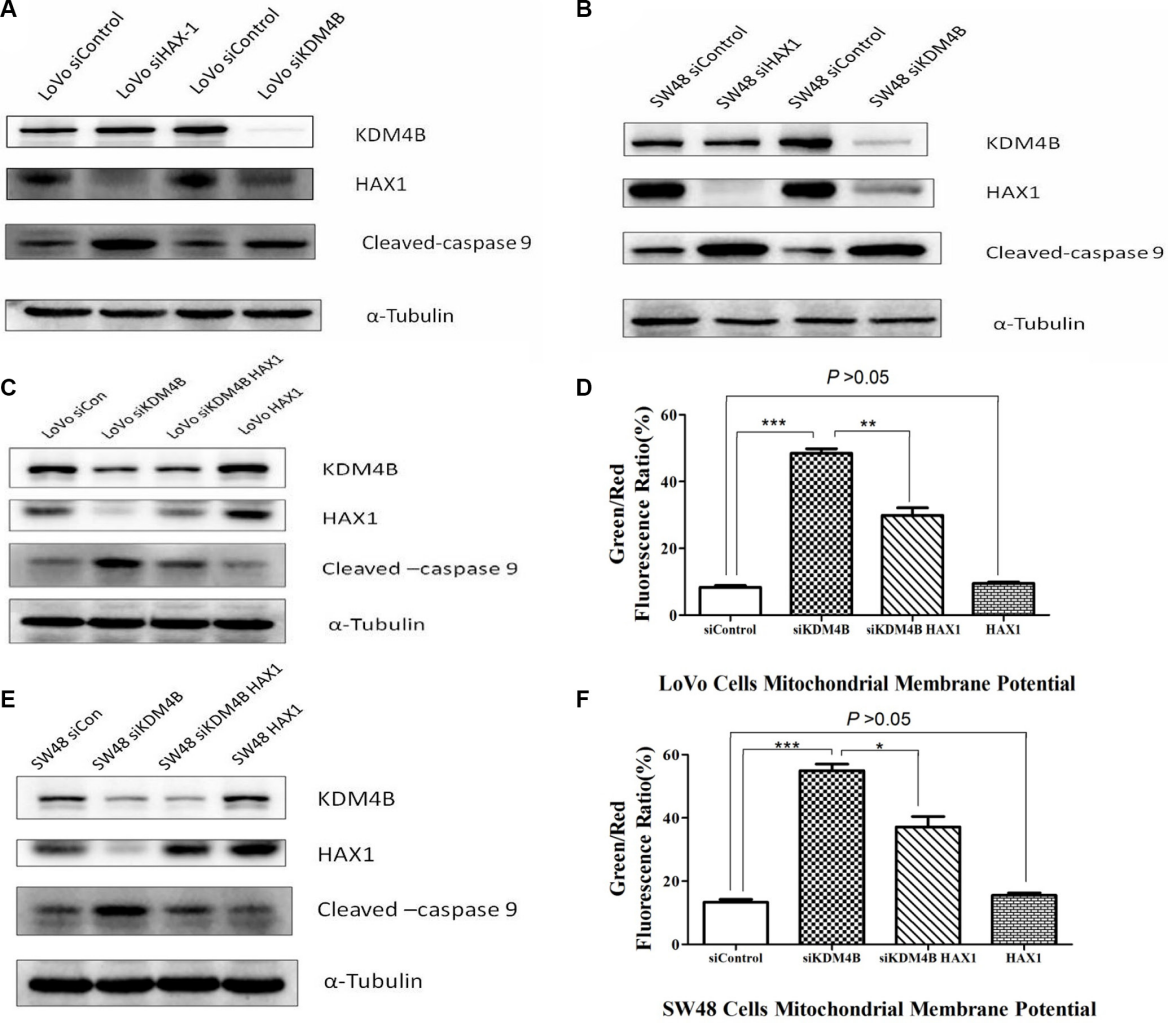
HAX1 is required for the role of KDM4B in mitochondrial apoptosis (**A** and **B**) Effect of KDM4B knockdown or HAX1 knockdown on levels of cleaved-caspase 9. LoVo cells (A) and SW48 cells (B) treated with siHAX1 or siKDM4B were examined by western blot analysis. (**C**, **D**, **G**, and **H**) Effect of HAX1 overexpression on mitochondrial apoptosis in stable KDM4B-depleted LoVo cells (C and D) and SW48 cells (**E** and **F**). Stable KDM4B-depleted cells transfected with control vectors or HAX1 plasmid for 48 h were examined by western blot analysis and JC-1 staining.

Previous reports have shown that HAX1 is an anti-apoptotic molecule that protects cardiac myocytes from hypoxia/reoxygenation-induced apoptosis, by inhibiting caspase-9 [22, 23]. To explore the relationship between KDM4B, HAX1, and caspase-9, we analyzed their expression following individual knockdown of KDM4B and HAX1 by siRNA. We observed that knockdown KDM4B led to a decrease in HAX1 expression and an increase in cleaved-caspase 9 in LoVo cells. In contrast, HAX1 knockdown enhanced the level of cleaved-caspase 9, but had no effect on KDM4B levels (Figure 5A). Similar results were also observed in SW48 cells (Figure 5B). To determine whether KDM4B regulates mitochondrial apoptosis in a HAX1-dependent manner, we overexpressed HAX1 in KDM4B stably silenced LoVo cells, and examined the level of cleaved-caspase 9. HAX1 overexpression restored the repression of cleaved-caspase 9 by KMD4B (Figure 5C). Next, we examined the effect of HAX1 overexpression on the mitochondrial apoptosis of KDM4B-depleted cells. In these cells, HAX1 overexpression partly restored the protective role of KDM4B in mitochondrial apoptosis (Figure 5D). These findings were also confirmed in SW48 cells (Figure 5E and 5F). Taken together, these results suggest that the role of KDM4B in mitochondrial apoptosis is dependent partly on HAX1.

### KDM4B is critical for colorectal cancer tumorigenesis in mouse xenograft models

**Figure 6:**
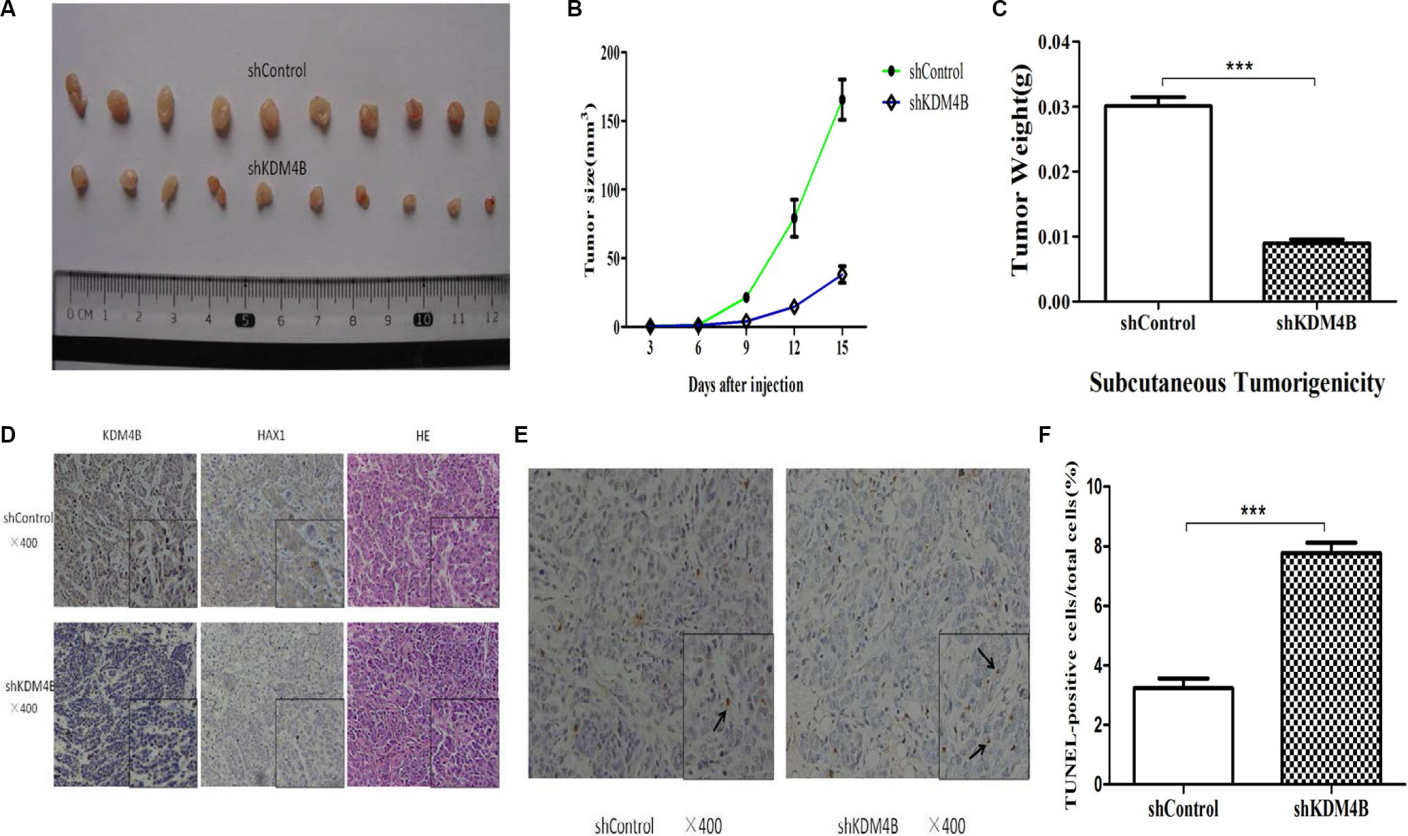
Tumorigenicity assays in nude mice (**A**) Stable KDM4B knockdown LoVo cells and control cells were injected subcutaneously into nude mice (*n* = 10 for each group). The tumors were collected and shown. (**B**) Tumor development was monitored for 15 days. The length and width of tumors were measured every 3 days to determine tumor volume (mean ± sd, *n* = 10). (**C**) The mean tumor weight of each group was calculated(shControl *vs* shKDM4B = 0.030 ± 0.004 *vs* 0.009 ± 0.002 g). (*P* < 0.001) (**D**) Representative images of immunohistochemical (IHC) staining (using anti-KDM4B and anti-HAX1 antibodies) and hematoxylin and eosin (HE) staining of tumor tissues were shown. (**E** and **F**) Representative images of TUNEL staining in tumor tissues are shown (E). The percentage of TUNEL-positive cells in total cells is shown (F) (shControl *vs* shKDM4B = 3.23 ± 0.57 *vs* 7.77 ± 0.61). (*P* < 0.001) The arrow indicates TUNEL-positive cell.

To address the role of KDM4B in colorectal cancer tumorigenesis *in vivo*, we generated stably KDM4B-depleted cell lines by transducing LoVo cells with KDM4B shRNA lentivirus. We next assessed tumor growth of KDM4B-depleted cell lines using a mouse xenograft model based on subcutaneous injection. Stable knockdown of KDM4B significantly repressed tumor growth compared with shControl cells (Figure 6A–6C).

In support of this, IHC analysis revealed that stably KDM4B-depleted cells expressed low levels of HAX1 and high TUNEL(+) levels, compared with tumors from shControl-treated cells (Figure 6D–6F). These results confirm not only the inhibitory effect of KDM4B knockdown on tumor growth, but also demonstrate that KDM4B knockdown promotes apoptosis.

### KDM4B and HAX1 expression are significantly correlated in colorectal tissues

**Figure 7:**
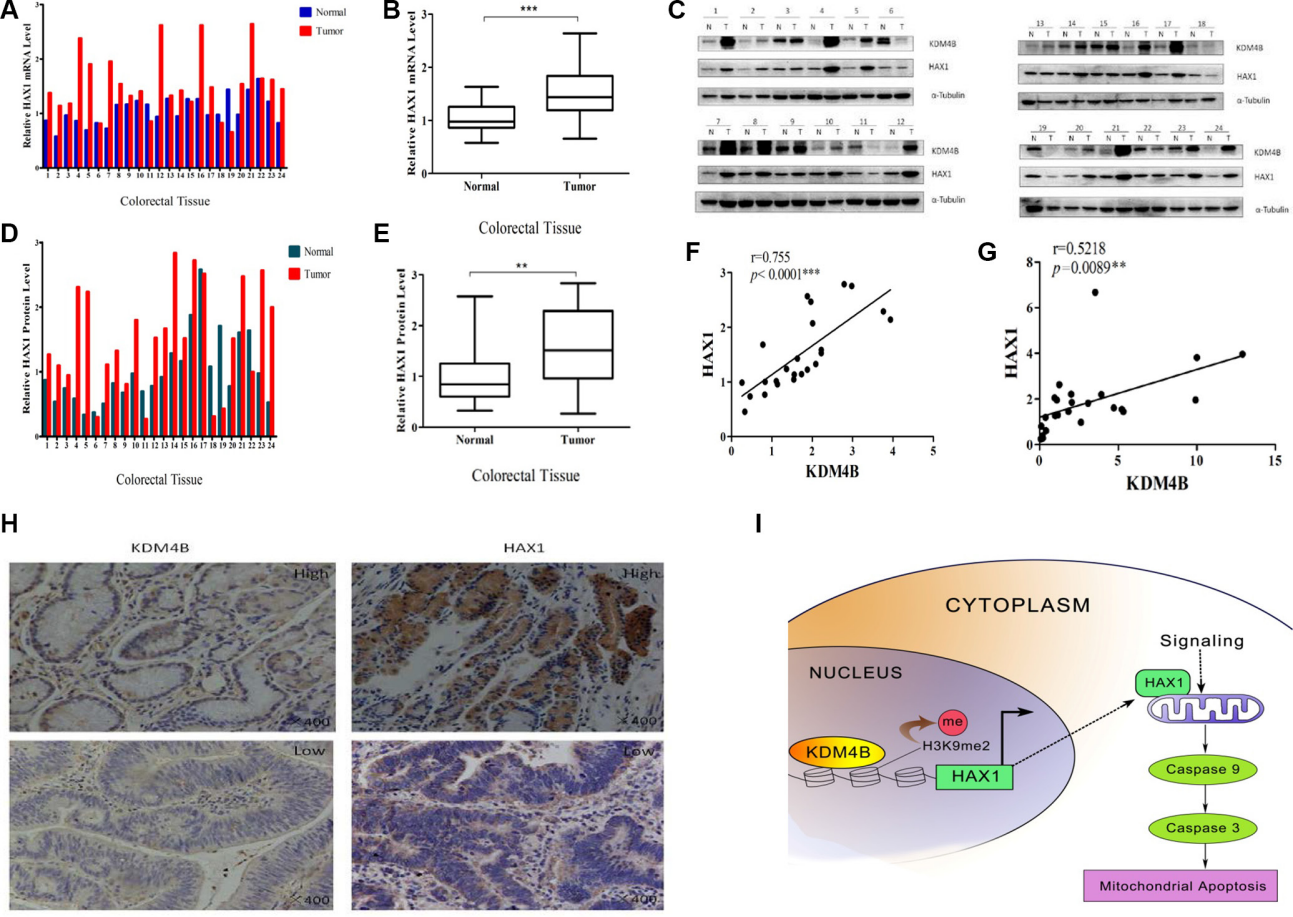
KDM4B and HAX1 expression are significantly correlated in colorectal tissues (**A**) Analysis of *HAX1* mRNA expression in colorectal tissues (including 24 colorectal tumor tissues and 24 corresponding colorectal normal tissues) by quantitative real-time PCR. (**B**) Quantitative real-time PCR results are shown in box-whisker plot. Student's *t* test (*P* < 0.001). (**C**) KDM4B and HAX1 protein levels in colorectal tissues (24 paired samples) were examined by western blot. (**D**) Quantification of HAX1 protein in colorectal tissues. Band intensities were measured using Image J, and normalized to α-tubulin. (**E**) Quantification of HAX1 protein is shown by box-whisker plot. For statistical analysis, Student's *t* test was adopted (*P* < 0.01). (**F** and **G**). Pearson *r* correlation was used to measure the relationship between KDM4B and HAX1 mRNA and protein levels. (F) *r* = 0.755, *P* < 0.0001; (G) *r* = 0.5218, *P* = 0.0089 (**H**) Representative images of IHC staining of CRC tumors with anti-KDM4B and anti-HAX1 antibodies. (**I**) A hypothetical representation of the regulatory pathway underlying KDM4B-induced cell mitochondrial apoptosis.

To compare expression levels of KDM4B and HAX1 in colorectal tissues, we performed quantitative RT- PCR and western blot analysis to examine the expression of HAX1 in the 24 primary colorectal tumor samples and 24 adjacent normal colorectal normal tissues. As expected, HAX1 was significantly overexpressed in CRC tissues at both mRNA and protein levels (Figure 7A–7E). We next analyzed the association between KDM4B and HAX1 expression using the Pearson r correlation test. We observed a significant positive correlation between KDM4B and HAX1 mRNA levels (Figure 7F, *r* = 0.755, *p* < 0.001) and protein levels (Figure 7G, *r* = 0.5218, *p* = 0.0089). These results were also confirmed by IHC in colorectal tissues (Figure 7H).

## DISCUSSION

Apoptosis is an essential process for organ homeostasis, by controlling cell number and tissue tropism under both physiological and pathological circumstances [24, 25]. Inappropriate activation of apoptosis underlies many common pathologies [26, 27]. Escape from apoptosis, or increased anti-apoptotic activities, play a key role in tumorigenesis. [28]. Mitochondria play a key role in cell death mechanisms, and cancer development is associated with suppression of apoptotic pathways [29]. Mitochondria are critical “gatekeepers” of apoptosis, and the regulatory mechanisms of mitochondrial apoptosis have substantially increased in complexity during the past decade as the center organelle of the intrinsic apoptotic pathways [30].

Within the past two decades, there has been increasing evidence of epigenetic dysregulation in multiple types of cancer, in which histone and DNA modification play a critical role in tumor growth and survival [31, 32]. Histone modifications play a causative role in oncogenesis. However, the role of histone demethylation in mitochondrial apoptosis remains unclear. In this study, we investigate the role of KDM4B in mitochondrial apoptosis. Our data demonstrate that KDM4B is involved in mitochondrial apoptosis and plays an important role in CRC cells (Figures 2 and 3).

During the course of our investigation into the mechanism by which KDM4B regulates mitochondrial apoptosis, we identified *HAX1*, an anti-apoptotic molecule, as a primary target of KDM4B-mediated gene activation (Figures 3 and 4). HAX1, a multi-functional protein, is ubiquitously expressed in various tissues, and expression has been reported in the mitochondria and the cytoplasm [33, 34]. Recently, HAX1 was reported as an anti-apoptotic molecule that protects cardiac myocytes from hypoxia/reoxygenation-induced apoptosis by inhibiting caspase-9 [22, 23]. HAX1 also participates in mitochondria-mediated apoptosis and functions as an endogenous anti-apoptotic molecule [35]. It is essential for maintenance of the MMP in myeloid and epithelial cells [36, 37]. Consistent with these reports, we previously demonstrated that HAX1 was critical for protecting cells against apoptosis [20]. In this study, KDM4B transcriptionally activated HAX1 expression and regulated methylation of H3K9me3 at the *HAX1* promoter (Figure 4). We further demonstrate that KDM4B inhibits mitochondrial apoptosis in a partly HAX1-dependent manner (Figure 5). Our clinical datas confirm that HAX1 expression is associated with KDM4B expression (Figure 7). These two genes are also overexpressed in CRC tissues compared with normal mucosa, indicating that aberrant expression of KDM4B and HAX1 may be related to carcinogenesis. Importantly, KDM4B knockdown inhibited tumor growth and increased the level of apoptosis in mouse xenograft tumors (Figure 6), indicating that KDM4B may be a potential target for therapeutic intervention.

Histone demethylases may be categorized into two classes based on their enzymatic mechanisms: the LSD family and the JmjC family [38]. Small-molecule inhibitors of the two families of histone demethylases are at various stages of development, and interest in such compounds has been spurred by emerging preclinical data showing the therapeutic potential of compounds that inhibit LSD1 in acute myeloid leukemia (AML) [39]. However, no specific small-molecule inhibitors of the KDM4 subfamily, important members of the JmjC family, have yet been reported. Our study may therefore provide an important basis for the development of small-molecule inhibitors targeting KDM4B.

It should be noted that we did not investigate the role of KDM4B in the extrinsic apoptotic pathway. Previous studies have shown that KDM4B also plays an important role in the extrinsic apoptotic pathway [40]; however, the specific mechanism remains unclear. It reminds us that the mechanism of KDM4B in cell apoptosis perhaps has other illustration owing to the complexity of apoptosis. Further studies are required to clarify this issue. In Sun BB et al.(J. Digestive Disease, 2014) study [40], KDM4B was involved in both intrinsic and extrinsic apoptotic pathway. But the detailed mechanism was revealed unclearly. Our study revealed a more detailed mechanism to elaborate the role of KDM4B in intrinsic apoptotic pathway.

In conclusion, our data support the notion that KDM4B transcriptionally activates HAX1 expression and inhibits methylation of H3K9me3 at the *HAX1* promoter (Figure 7I). Thus, our study reveals a previously unidentified mechanism by which a histone methylation modifier is primarily involved in mitochondria-mediated apoptosis, by epigenetic regulation of *HAX1*. These results indicate that KDM4B may be an ideal target for cancer therapy. Further studies are necessary to investigate the potential therapeutic benefit of targeting KDM4B in CRC and for the development of novel epigenetic drugs targeting this enzyme.

## MATERIALS AND METHODS

### Cell culture

All colorectal cancer cell lines (LoVo, SW48, SW480, and SW620) were obtained from the Type Culture Collection cell bank (Chinese Academy of Sciences, Beijing, PR China). Cells were cultured at 37°C, 5% CO_2_ in Dulbecco Modified Eagle Medium (DMEM), supplemented with 10% fetal bovine serum (Hyclon; Thermo Scientific, Rockford, IL, USA).

### Tissue samples

Paired surgical specimens of colorectal tissues were collected from 24 patients with CRC. Tumor tissue and adjacent normal colorectal tissue were collected from primary resections of colorectal tumors from the same patient, and snap frozen in liquid nitrogen. The nature of tumor specimen was in Supplementary Table S1. This study was approved by the Huazhong University of Science and Technology Research Ethics Committee.

### mRNA isolation and real-time PCR assay

Total mRNA was extracted using TRIzol regent (Invitrogen, Carlsbad, CA, USA) and reverse transcription was performed using an RT-PCR kit (Fermentas, Beijing, China). Real-time experiments were conducted on an ABI Prism 7300 Real-time PCR system (Applied Biosystems, Foster City, CA, USA) using Maxima SYBR Green qPCR Master Mix (Thermo Scientific, Rockford, IL, USA). Relative gene expression was normalized to *18s* rRNA expression. The primer sequences for qRT-PCR are listed in Supplementary Table S2. qRT-PCR was performed in technical triplicate, and experiments were repeated at least three times.

### Western blot analysis

Total cells and tissues were extracted using NP-40 lysis buffer in the presence of a protease inhibitor cocktail (KeyGEN, Nanjing, China). Proteins were resolved on sodium dodecyl sulfate–polyacrylamide gel electrophoresis and transferred to polyvinylidene difluoride membranes (Immobilon; Millipore, Merck KGaA, Darmstadt, Germany). Membranes were probed with anti-KDM4B (8639; Cell Signaling Technology), anti-HAX1 (sc-166845; Santa Cruz Biotechnology), anti-cleaved caspase-9 (7237; Cell Signaling Technology), anti-cleaved caspase-3 (9664; Cell Signaling Technology), anti-histone H3 (ab1791; Abcam), anti-H3K9me3 (9754S; Cell Signaling Technology), anti-α-tubulin (12152; Cell Signaling Technology), anti-Bcl-2 (2870; Cell Signaling Technology), and anti-Bax (2772; Cell Signaling Technology) antibodies. Protein bands were incubated with HRP-conjugated antibodies and visualized using electrochemiluminescence by a chemiluminescence instrument.

### RNA interference

siRNA duplexes targeting the human *KDM4B* gene (siKDM4B) and *HAX1* gene (siHAX1) were synthesized and purified by RiboBio (Ribobio, Guangzhou, China). siRNA duplexes with non-specific sequences were used as an siRNA-negative control. RNA oligonucleotides were transfected using Lipofectamine RNAiMAX Reagent (Invitrogen) and the expression levels of KDM4B and HAX1 were quantified 72 h after transfection.

### Expression plasmid constructs

cDNA constructs encoding KDM4B or Flag were cloned into the pcDNA3.1 expression vector using standard cloning methodology. Eukaryotic expression plasmids (2 ug) expressing KDM4B or Flag were transfected into LoVo cells in 6-cm dishes using 10 μL Lipofectamine 2000 (Invitrogen). Cells were harvested 72 h post-transfection for further analysis.

### Cell apoptosis assays

Cell apoptosis was assessed by Annexin V-FITC and PI staining. Cells were stained with Annexin V-FITC and PI, in the dark for 20 min at room temperature, and analyzed by flow cytometry (FACSCalibur; BD Biosciences, Franklin Lakes, NJ, USA).

### Measurement of mitochondrial membrane potential

Mitochondrial membrane potential was measured using a fluorescent probe, JC-1. JC-1 is a cationic dye that exhibit potential-dependent accumulation in mitochondria. At low mitochondrial membrane potential, JC-1 is a green-fluorescent monomer, whereas at higher potential, JC-1 becomes aggregate and emits red fluorescence. JC-1 can be used as an indicator of mitochondrial potential. The collapse of mitochondrial membrane potential is the early stage of apoptosis. The ratio of JC-1 green to red fluorescence reflects the ratio of apoptosis. Cells were cultured under various experimental conditions and subsequently incubated with JC-1 dye diluted in culture medium at 37°C for 20 min. Cells were then immediately analyzed by flow cytometry (FACSCalibur; Biosciences, Franklin Lakes, NJ, USA).

### Terminal deoxynucleotidyl transferase-mediated dUTP-biotin nick end labeling (TUNEL) assays

TUNEL assays were performed to evaluate apoptosis. TUNEL staining was performed on transiently transfected colorectal cancer cells following immediate fixation in 4% paraformaldehyde for 30 min, or on colorectal cancer tissue sections (4 um), in accordance with the manufacturer's instructions (*In Situ* Cell Death Detection Kit, Roche Diagnostics). Randomly selected microscopic fields (*n* = 5) were evaluated to calculate the apoptotic rate.

### Chromatin immunoprecipitation (ChIP) assays

ChIP assays were performed in accordance with the manufacturer's protocols (EpiQuik Chromatin Immunoprecipitation Kit, Epigentek Group Inc.), and repeated at least three times. To examine changes in KDM4B-binding activity at the *HAX1* promoter, ChIP assays were performed with an anti-KDM4B antibody (8639; Cell Signaling Technology), using the Millipore ChIP assay protocol. In brief, cells were cross-linked with 1% formaldehyde for 10 min at 37°C, collected in SDS lysis buffer, and DNA was fragmented (200–1000 bp) by sonication. Antibodies against KDM4B or control were added to each aliquot of pre-cleared chromatin and incubated overnight. Protein G-agarose beads were added and incubated for 2 h at 4°C. After a series of washes, cross-linking was reversed and DNA was extracted and purified for PCR. The primer sequences for ChIP are listed in Supplementary Table S2. PCR values were normalized to input and calculated as percentage of input. To examine the status of H3K9 methylation, ChIP assays were performed using anti-H3K9me3 antibody (9754S; Cell Signaling Technology) as described above.

### Luciferase assays

Luciferase activity assays were performed using the Dual-Luciferase Reporter Assay System (Promega, Madison, WI, USA), in accordance with the manufacturer's protocol. Cells were seeded in 24-well plates and *HAX1* promoter luciferase reporter constructs (100 ng) were transfected with siKDM4B (100 nM) or KDM4B plasmid (2 ug) and siControl (100 nM) or Control plasmid (2 ug). Luciferase assays were performed 72 h after transfection using the Dual-Luciferase Assay System. Firefly luciferase activity was normalized to the corresponding Renilla luciferase activity. All experiments were performed three times.

### Cell viability assays

Cell viability was evaluated using the Cell Counting Kit 8 (CCK-8; Dojindo, Kumamoto, Japan) according to manufacturer's instructions. Cells (4000 cells/well) were seeded in 96-well plates in 100 μL culture medium and subject to various experimental treatments. CCK-8 solution was then added to each well (10 μL) and plates were incubated at 37°C for 2 h. OD was measured at 450 nm using a Microplate Reader (Sunrise, TECAN, Männedorf, Switzerland). All experiments were performed in triplicate.

### Generation of stable KDM4B-depleted cells

KDM4B was depleted in colorectal cancer cells using a KDM4B lentiviral construct expressing shKDM4B (Genechem, Shanghai, China). Following infection with lentivirus, cells were expanded in medium containing puromycin (1.5 μg/mL), and screened for KDM4B knockdown by western blot analysis. Empty vector-infected cells were used as a control.

### Mouse tumor xenograft

For the *in vivo* xenograft study, stably transduced LoVo cells were used. 5 × 10^6^ stable KDM4B-depleted or shControl LoVo cells in 200 μL of Matrigel (BD Biosciences, Franklin Lakes, NJ, USA) were injected subcutaneously into athymic female nude mice. Ten animals per group were used in each experiment. Following their visual appearance, tumors were measured every 3 days using a vernier caliper. At a defined timepoint, all mice were sacrificed and tumors were collected and weighed. Tumor volume was calculated using the formula: tumor volume (mm^3^) = 0.5 × length × width^2^ and a tumor growth curve was drawn.

### Immunohistochemistry

Immunohistochemistry was performed on tissue microarrays containing 90 paired colorectal adenocarcinoma and corresponding normal colorectal epithelium specimens to compare *in situ* expression of KDM4B and HAX1. Slides were incubated with antibody against KDM4B (1:100, A301-478A, Bethyl Laboratories, Montgomery, TX, USA) or HAX1 (1:200, sc-166845, Santa Cruz, CA, USA) accordance with the manufacturer's protocol.

### Statistics

KDM4B or HAX1 mRNA or protein levels were compared using the Student *t* test. The Pearson r correlation test was used for correlation analysis. Data were analyzed using SPSS 19.0 software. Two groups of data were statistically analyzed by two-tailed *t* tests using Graphpad Prism 5 Software. *P* < 0.05 was considered statistically significant.

## SUPPLEMENTARY MATERIALS FIGURES AND TABLE


