# Supplementary material for: KDM4B plays an important role in mitochondrial apoptosis by upregulating HAX1 expression in colorectal cancer
Source: Oncotarget. 2016 Aug 5;7(36):57866–77. doi: 10.18632/oncotarget.11077 (PMC5295396; doi:10.18632/oncotarget.11077)
Supplement: Supplementary file 1 [file oncotarget-07-57866-s001.pdf]

KDM4B plays an important role in mitochondrial apoptosis by upregulating HAX1 expression in colorectal cancer

Supplementary Materials

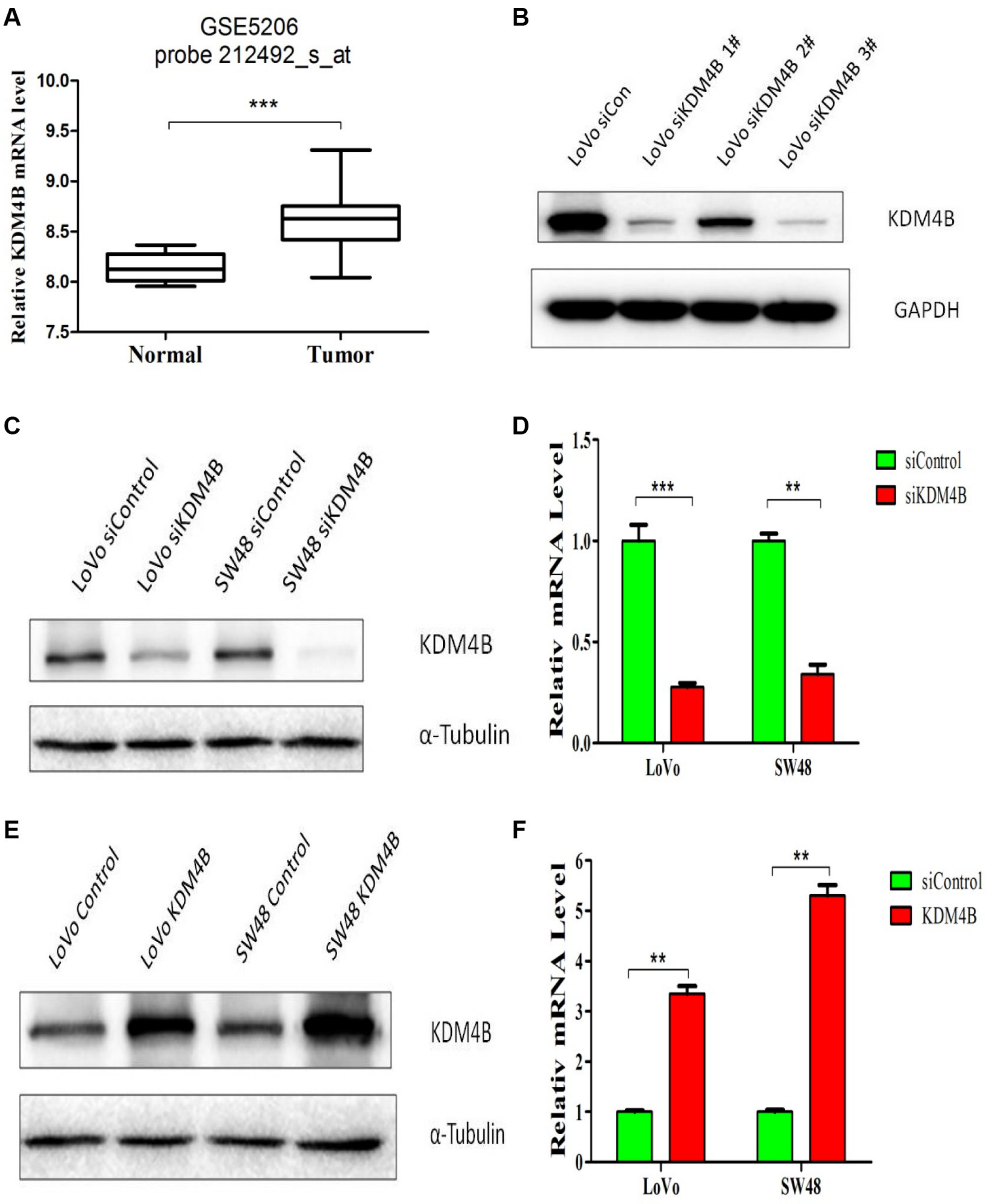

Supplementary Figure S1: (**A**) Expression of KDM4B in GEO dataset (GSE5206), ***P* < 0.05.** (**B**) Expression of KDM4B in LoVo cells with 3 siRNA duplexes targeting KDM4B for 72 h by western blot. (**C** and **D**) Expression of KDM4B protein and mRNA was analyzed following treatment of LoVo cells and SW48 cells with siRNA(1#) or siControl for 72 h, by western blot and quantitative real-time PCR, respectively. (**E** and **F**) Expression of KDM4B protein and mRNA was analyzed following transfection of LoVo cells and SW48 cells with KDM4B plasmid or empty vector for 48 h, by western blot and quantitative real-time PCR, respectively.

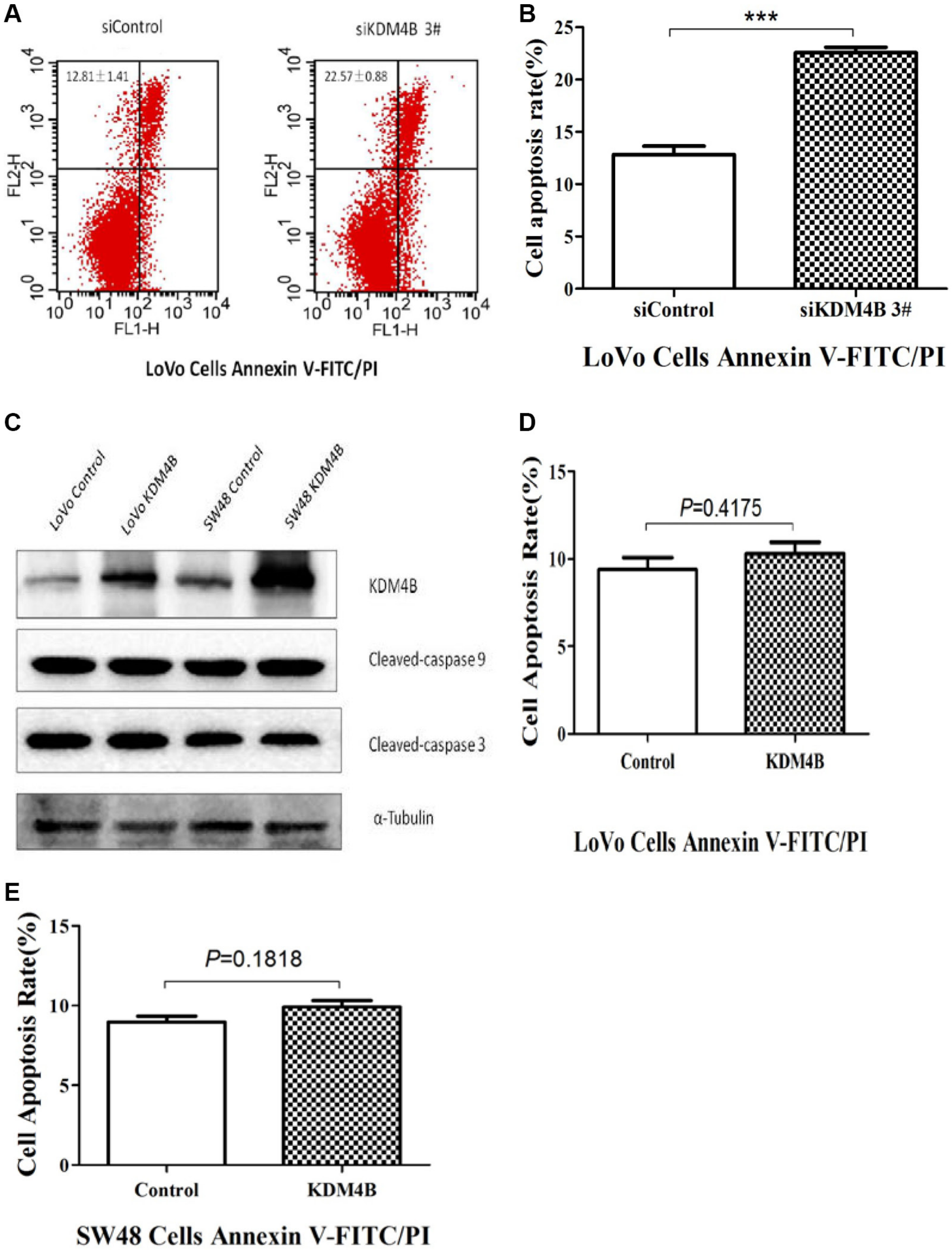

Supplementary Figure S2: (**A** and **B**) Knockdown of KDM4B induces significant apoptosis in LoVo cells by Annexin V-FITC/PI staining analysis. LoVo cells were treated with siControl(12.81 ± 1.41) or siKDM4B(3#,22.57 ± 0.88) for 72 h, then cell apoptosis was tested by Annexin V-FITC/PI staining analysis.(B, $P < 0.05$). (**C**) Effect of KDM4B overexpression on levels of cleaved-caspase 9 and cleaved-caspase 3. LoVo cells and SW48 cells were transfected with KDM4B plasmid or empty vector for 48 h and cleaved-caspase 9 and cleaved-caspase 3 levels were examined by western blot. (**D** and **E**) KDM4B overexpression had no significant effect on apoptosis in LoVo cells and SW48 cells by Annexin V-FITC/PI staining analysis ($P > 0.05$).

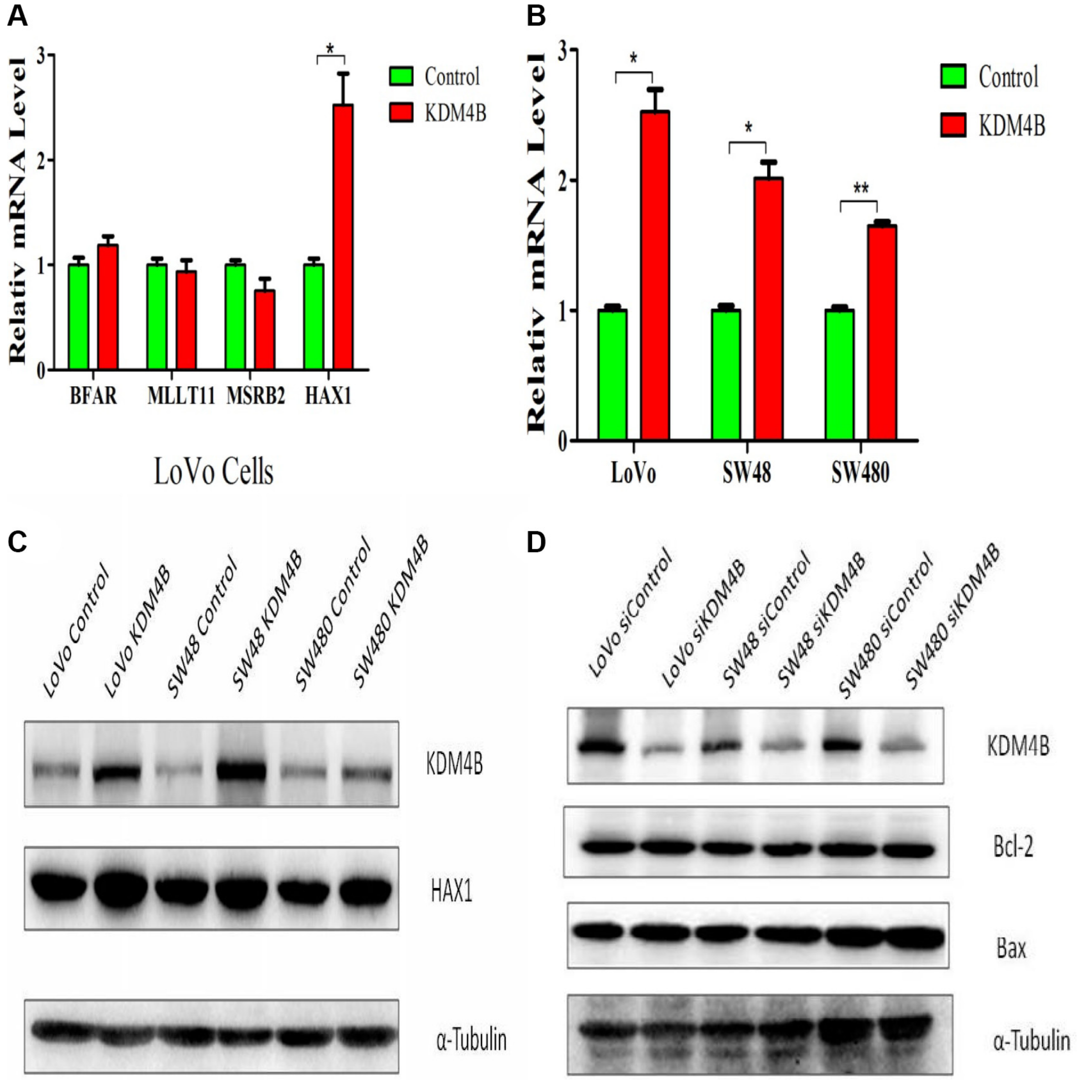

Supplementary Figure S3: (**A**) Expression levels of *BFAR, MLLT11, MSRB2,* and *HAX1* in LoVo cells following transfection with KDM4B plasmid for 48 h. mRNA levels were analyzed by quantitative RT-PCR. (**B** and **C**) Expression levels of HAX1 in colorectal cancer cells (LoVo, SW48, SW480) following transfection with KDM4B plasmid for 48 h by RT-PCR and western blot. (**D**) KDM4B knockdown had minimal effect on the expression of Bcl-2 and Bax. Colorectal cells (LoVo, SW48, SW480) were treated with siRNA targeting KDM4B or negative control for 72 h. The expression of Bcl-2 and Bax were analyzed by western blot.

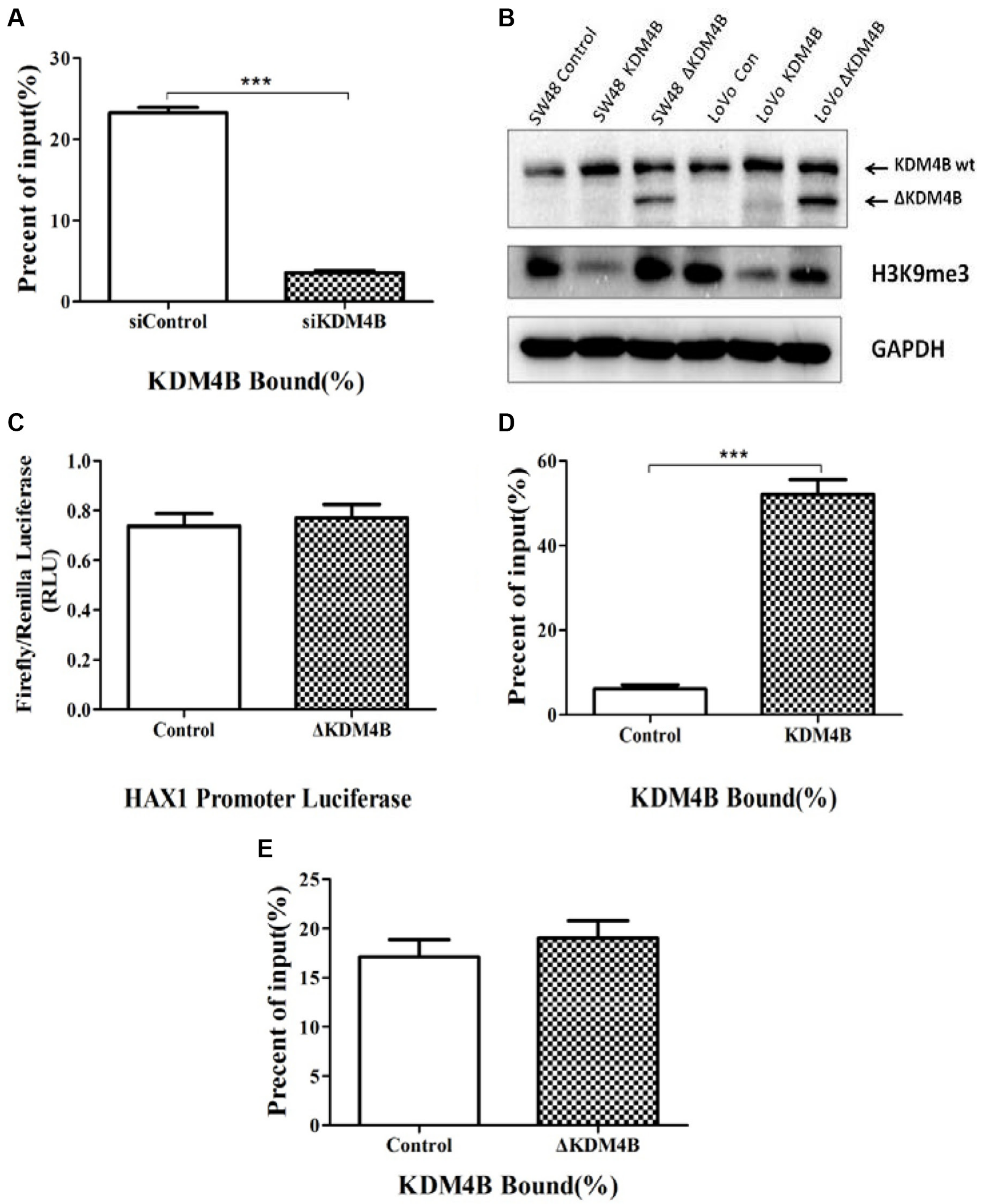

Supplementary Figure S4: (**A**) Quantification by qRT-PCR of *HAX1* promoters from anti-KDM4B ChIP samples. LoVo cells were treated with siKDM4B (1#) for 72 h prior to ChIP analysis using an anti-KDM4B antibody. Analysis of KDM4B recruitment to the *HAX1* promoter was assessed by quantitative RT-PCR. The result is shown as a percentage of the input chromatin. (**B**) Expression of KDM4B and H3K9me3 in LoVo cells following transfection with KDM4B or ΔKDM4B plasmid for 48 h. (**C**) LoVo cells were transfected with ΔKDM4B plasmid and the *HAX1* promoter plasmid. Relative luciferase activity was assayed. $P > 0.05$. (**D** and **E**) Quantification by qRT-PCR of *HAX1* promoters from anti-KDM4B ChIP samples. LoVo cells were treated with KDM4B orΔKDM4B plasmid for 48 h prior to ChIP analysis using an anti-KDM4B antibody. Analysis of KDM4B recruitment to the *HAX1* promoter was assessed by quantitative RT-PCR. The result is shown as a percentage of the input chromatin.

Supplementary Table S1: Oncologic data

Tumor size (cm)	5.2*
T-stage (n)	
T *in situ*	0
T1	0
T2	3
T3	16
T4	5
N-stage (n)	
N0	15
N1	7
N2	2
No. of retrieved nodes	19*
M-stage (n)	
M0	24
M1	0
WHO differentiation grade (n)	
1	1
2	14
3	9
4	0

*Median W*HO* World Health Organization

Supplementary Table S2: Primer sequences for quantitative RT-PCR

Gene Name	Primer Sequence
18s-f	GGAATTGACGGAAGGGCACCACC
18s-r	GTGCAGCCCCGGACATCTAAGG
KDM4B-f	AGACGTATGATGACATCGACGA
KDM4B-r	CGTAGATCGGGGAGACAAAGG
HAX1-f	CAGGAGGAGGGATACGTTTCC
HAX1-r	CCCATATCGCTGAAGATGCTATT
BFAR-f	GGCCCTCAGATTTCTGTTAGTG
BFAR-r	TGTGCCCACAGTTCAAGGTG
MLLT11-f	GGACCCTGTGAGTAGCCAGTA
MLLT11-r	CAGCTCCGACAGATCCAGT
MSRB2-f	CGGAGCAGTTCTACGTCACAA
MSRB2-r	CAGCACACGCAATGATACATTC
HAX1-ChIP-f	GCGTCATGAATATGAACAGCA
HAX1-ChIP-r	GTCTTAGGGGTGAGGGGAAG

Supplementary Table S3: siRNA sequences

siRNA Name	Sequence
siHAX1	GAAUUUGGCUUCGGCUUCA
siKDM4B 1#*	GCGCAGAAUCUACCAACUU
siKDM4B 2#	CAAAUACGUGGCCUACAUA
siKDM4B 3#	CGGCCACAUUACCCUCCAA

*Yang J, Jubb AM, Pike L, Buffa FM, Turley H, Baban D, Leek R, Gatter KC, Ragoussis J and Harris AL. The histone demethylase JMJD2B is regulated by estrogen receptor alpha and hypoxia, and is a key mediator of estrogen induced growth. Cancer research. 2010; 70(16):6456-6466.
